# Azithromycin Reduction to Reach Elimination of Trachoma (ARRET): study protocol for a cluster randomized trial of stopping mass azithromycin distribution for trachoma

**DOI:** 10.1186/s12886-020-01776-4

**Published:** 2021-01-06

**Authors:** Abdou Amza, Boubacar Kadri, Beido Nassirou, Ahmed M. Arzika, Ariana Austin, Fanice Nyatigo, Elodie Lebas, Benjamin F. Arnold, Thomas M. Lietman, Catherine E. Oldenburg

**Affiliations:** 1 Programme National de Santé Oculaire, Ministere de la Santé Publique, Niamey, Niger; 2 Centre de Recherche et d’Intervention en Santé Publique, Niamey, Niger; 3 Francis I Proctor Foundation, University of California, San Francisco, USA; 4 Department of Ophthalmology, University of California, 490 Illinois St, Floor 2, San Francisco, CA 94158 USA; 5 Department of Epidemiology & Biostatistics, University of California, San Francisco, USA

**Keywords:** Trachoma, Azithromycin, Mass drug administration, Neglected tropical diseases

## Abstract

**Background:**

The World Health Organization (WHO) recommends annual mass azithromycin distribution until districts drop below 5% prevalence of trachomatous inflammation—follicular (TF). Districts with very low TF prevalence may have little or no transmission of the ocular strains of *Chlamydia trachomatis* that cause trachoma, and additional rounds of mass azithromycin distribution may not be useful. Here, we describe the protocol for a randomized controlled trial designed to evaluate whether mass azithromycin distribution can be stopped prior to the current WHO guidelines.

**Methods:**

The Azithromycin Reduction to Reach Elimination of Trachoma (ARRET) study is a 1:1 community randomized non-inferiority trial designed to evaluate whether mass azithromycin distribution can be stopped in districts with baseline prevalence of TF under 20%. Communities in Maradi, Niger are randomized after baseline assessment either to continued annual mass azithromycin distribution or stopping annual azithromycin distribution over a 3-year period. We will compare the prevalence of ocular *C. trachomatis* (primary outcome), TF and other clinical signs of trachoma, and serologic markers of trachoma after 3 years. We hypothesize that stopping annual azithromycin distribution will be non-inferior to continued annual azithromycin distributions for all markers of trachoma prevalence and transmission.

**Discussion:**

The results of this trial are anticipated to provide potentially guideline-changing evidence for when mass azithromycin distributions can be stopped in low TF prevalence areas.

**Trial registration number:**

This study is registered at clinicaltrials.gov (NCT04185402). Registered December 4, 2019; prospectively registered pre-results.

## Background

Annual mass azithromycin distribution is a cornerstone of the World Health Organization (WHO)’s trachoma control strategy and leads to rapid reduction in the prevalence of ocular *Chlamydia trachomatis*, the causative organism of trachoma [1–3]. Mass azithromycin distribution is indicated in evaluation units (roughly equivalent to a health district) with at least 10% prevalence of trachomatous inflammation—follicular (TF), a clinical sign of active trachoma. Evaluation units receive 3 to 5 years of annual mass azithromycin distribution, at which point trachoma impact surveys are conducted to re-assess if continued intervention is required. For evaluation units falling below 5% TF, annual mass azithromycin distribution is discontinued. The 10% and 5% TF thresholds are based on expert consensus and have not been empirically validated. Whether earlier discontinuation of azithromycin distribution would lead to similar outcomes or increase the probability of recrudescence is unknown.

The clinical sign of trachoma used for surveillance, TF, is relatively easy to use via field grading and does not require a cold chain or access to a laboratory, as would be required for surveillance of ocular chlamydia infection. TF occurs as part of the inflammatory response to ocular chlamydia infection and is a lagging indicator for ocular chlamydia infection. At the community level, TF prevalence declines more slowly in the presence of mass azithromycin distribution than ocular chlamydia infection prevalence [4]. Districts with TF prevalence above the threshold may have very low or zero prevalence of ocular chlamydia infection, and thus additional rounds of azithromycin distribution may not be helpful for eliminating disease.

The Azithromycin Reduction to Reach Elimination of Trachoma (ARRET) study was designed to test whether discontinuation of annual mass azithromycin distribution is non-inferior to continued annual mass azithromycin distribution after three years in districts with up to 20% prevalence of TF. We hypothesize that the prevalence of ocular chlamydia infection, the target of azithromycin distribution, is sufficiently low in these communities that additional rounds of azithromycin distribution will not lead to reduce ocular chlamydia or TF prevalence compared to stopping distribution.

## Methods/design

### Study design

ARRET is a cluster randomized trial designed to evaluate whether stopping mass azithromycin distribution is non-inferior to continued annual mass azithromycin in districts with TF prevalence below 20% in Maradi, Niger (Table 1). *Grappes*, which are government-defined communities approximately the size of a village, in evaluation units in Maradi that have TF prevalence below 20% during the last trachoma impact survey are randomized in a 1:1 fashion to stopping annual mass azithromycin distribution or continuing distribution for 3 years. The primary outcome is the prevalence of ocular *C. trachomatis* infection after 36 months.

**Table 1:** SPIRIT diagram of study assessments

TIMEPOINT	Baseline	Allocation	Follow-up		
		Mo. 0	***Mo. 12***	***Mo. 24***	***Mo. 36***
**ENROLMENT:**					
**Community eligibility screen**	X				
**Informed consent**	X				
**Census**	X		X	X	X
**Allocation**		X			
**INTERVENTIONS:**					
***Annual Azithromycin MDA***		X	X	X	(X)^a^
***No treatment***		X	X	X	(X)^a^
**ASSESSMENTS:**					
***Conjunctival swabs***	X				X
***Conjunctival photography***	X				X
***Dried blood spot collection***	X				X

^a^Communities will receive mass drug administration with azithromycin per Niger trachoma program guidelines at and after the 36-month study visit, depending on the prevalence of trachomatous inflammation--follicular

### Objective and hypothesis

The overall objective of this study is to determine if annual mass azithromycin distribution can be stopped before the current 5% TF prevalence guideline in communities with low prevalence of TF. We hypothesize that discontinuing annual mass azithromycin distribution in communities within districts with TF prevalence between 5 and 20% will be non-inferior to continuing mass azithromycin distribution. We further hypothesize that the prevalence of ocular *C. trachomatis* in these communities will be very low and the confidence interval will include zero.

### Study oversight

An independent Data and Safety Monitoring Committee (DSMC) oversees this study. The DSMC includes members with expertise in ophthalmology, infectious diseases (including trachoma specifically), biostatistics, epidemiology, clinical trials, and ethics. The DSMC meets annually in a face-to-face meeting, including once prior to study initiation to review and approve study procedures, and then for ongoing monitoring annually. Aggregate quarterly reports with study data are submitted to the DSMC for monitoring during periods of active data collection.

### Study setting

This study will take place in 80 communities in one district of Maradi Region, Niger. Annual mass azithromycin distribution began in 2008. TF prevalence in the study district during the most recent trachoma impact survey (2018) was 13.5%.

### Community recruitment and eligibility

All communities in the study district are eligible for inclusion in the study. Consent for individual communities to participate in the study is obtained from local community leaders. Communities are eligible to participate if they are in the study district regardless of individual TF prevalence.

### Randomization

Communities are randomized to stopping mass azithromycin distribution or continued annual mass azithromycin distribution per the national trachoma program’s normal protocol after the baseline assessment. Communities are randomized to stopping or continuing azithromycin for three years in a 1:1 allocation without blocking or stratification. Allocation concealment is achieved by randomizing after baseline assessments are complete and assigning all communities to an intervention at the same time.

### Census

Prior to baseline and 36-month monitoring visits, a door-to-door enumerative census is undertaken in each study community. The census includes the composition of each household, and all members residing in all households in the study communities are recorded. Census data are collected on the study’s mobile application on a tablet, and includes the name, gender, and age of each household member and GPS coordinates of the physical structure of the household.

### Monitoring

Monitoring occurs in all study communities at baseline and 36 months after baseline. A random sample of 50 children aged 0 to 9 years will be monitored in each study community for ocular *C. trachomatis*, clinical signs of trachoma, and serologic markers of exposure to *C. trachomatis*. A conjunctival swab will be collected from each child randomly selected for monitoring by everting the right upper eyelid and swabbing the tarsal conjunctiva three times, rotating the swab with each pass. Swabs will be processed with quantitative PCR pooled in groups of 5 as previously described [5]. Laboratory personnel will be masked to the community of origin of the swabs and swabs will be processed in a random order. Conjunctival photography will be used to measure active trachoma. We will use a smartphone camera fitted with a CellScope attachment to magnify the image [6, 7]. All images are graded in a grading center by standardized graders in a random order, assessing photographs for presence of TF or TI per the WHO simplified grading scale [8]. Dried blood spots will be collected in a subsample of 40 children to measure serologic markers of trachoma transmission with a finger or heel stick and collected on filter paper.

### Interventions

Communities are randomized to either stopping mass azithromycin distribution for the 36-month duration of the study or continued annual mass azithromycin distribution per Nigerien trachoma program guidelines (a total of 3 annual mass azithromycin distributions). After the final 36-month monitoring visit, communities in both arms will re-enter the national trachoma control program and will receive mass azithromycin distribution if indicated.

### Masking

Due to the nature of the intervention, communities are not masked. Study team members will not be made aware of which study arm a community is in and will not be involved in the mass drug administration program in communities continuing azithromycin distribution. All outcome assessments will be masked, as laboratory personnel processing conjunctival swabs and dried blood spots and graders scoring conjunctival photographs will be masked to community of origin. Swabs, dried blood spots, and photographs will be processed in a random order.

### Data collection and management

Data are collected electronically in the field using a mobile phone application hosted by CommCare (Dimagi Inc, Cambridge, MA, USA). Data are synced at least weekly or more often if an internet connection is available. Data are stored on encrypted servers to minimize risk of loss of confidentiality. No identifiable data will be shared outside of the study team.

### Primary outcome

The primary outcome is the community-level prevalence of ocular *C. trachomatis* measured at the 36-month study visit. Swabs from the same age stratum and community are processed in pools of 5 random swabs to increase efficiency.

### Secondary outcomes

Secondary outcomes include C. trachomatis load from quantitative PCR, community-level seroprevalence of C. trachomatis antigens Pgp3 and CT694 measured in dried blood spots [9], and community-level prevalence of the clinical signs of trachoma TF and TI as assessed by conjunctival photography.

### Adverse events

Adverse events following mass azithromycin distribution are rare and are mainly gastrointestinal (e.g., nausea or diarrhea) [10–12]. Adverse event data will be collected as in trachoma control programs. Participants are informed about the possibility of experiencing adverse reactions and that they are not serious. During each treatment round, participating households are asked to report any serious adverse events to program staff, who report it to the national trachoma control program manager and study staff.

### Sample size considerations

The sample size for ARRET is based on the primary outcome, the community-level prevalence of ocular C. trachomatis, under a non-inferiority trial design. Assuming a standard deviation of 0.05 (in absolute proportion) for the community-level prevalence of ocular *C. trachomatis *[13] and a correlation between baseline and 36-month outcomes of 0.5, we estimated an effective standard deviation of 0.043. Under these assumptions, a sample size of *N* = 80 communities (40 per arm) will provide at least 80% power to detect a non-inferiority margin of 3% [14].

### Statistical analysis

For the primary outcome, we will estimate the difference in community-level ocular C. trachomatis prevalence among children 0–9 years of age between arms using a linear regression model adjusting for baseline prevalence and including the community’s randomized treatment arm as a covariate. The analysis will be performed at the community level due to the community randomized nature of the intervention, and thus the analysis will account for community level clustering. A square-root transformation will be used for infection prevalence if necessary to improve normality and heteroskedasticity. The pre-specified non-inferiority margin is 3% prevalence. We will estimate a two-sided 95% confidence interval for the prevalence difference. If the upper 95% confidence interval of the prevalence difference (stopping azithromycin distribution – continued distribution) is < 3% and the lower 95% confidence interval includes zero, we will determine that stopping is non-inferior to continuing azithromycin distribution.

Secondary outcomes will be analyzed similarly for dichotomous outcomes (e.g., inflammation and active trachoma, seropositivity to Pgp3 and CT-694) to the primary outcome. Continuous secondary outcomes (e.g., chlamydia infectious load) will be modeled using a clustered regression model (clustering on community) using baseline community load and treatment arm as covariates. Uninfected individuals have a chlamydia load of zero, and we will model this with a Bernoulli-gamma mixture model (zero-inflated gamma distribution).

### Interim analysis

Because monitoring only occurs at baseline and 36 months after trial implementation, there are no planned interim analyses.

### Dissemination plan

Results of this study will be disseminated to policymakers in Niger, other trachoma-endemic countries, and the World Health Organization as well as locally in participating communities. Results will be presented at national and international conferences and published in peer-reviewed journals.

## Discussion

The results of this study are expected to generate potentially policy-changing results related to when to stop mass azithromycin distribution for trachoma control. Currently, WHO recommends continued annual mass azithromycin distribution until district-level prevalence of TF reduces below 5%. However, azithromycin is effective against the ocular strains of *C. trachomatis* that cause trachoma and not clinical signs of inflammation directly. If communities with low prevalence of TF do not have ongoing transmission of ocular *C. trachomatis*, continued azithromycin distribution will not likely yield additional benefits for trachoma control. Some evidence has suggested that continued mass azithromycin distribution may not be beneficial in communities with 5 to 9.9% prevalence of TF [15]. Additional evidence that continued azithromycin distribution in communities with higher TF prevalence (above the 10% threshold) does not lead to additional reduction in ocular chlamydia transmission would aid with decision-making relating to policy for mass azithromycin distribution. If azithromycin distributions could be stopped in lower prevalence communities, this may allow for refocusing of resources towards areas with persistently high trachoma prevalence despite many years of intervention [16].

Evidence for when to stop mass azithromycin distribution for trachoma control may also help to limit distribution of unnecessary antibiotics, which could reduce some antibiotic selection pressure and reduce macrolide resistance. Mass distribution of azithromycin has been shown to select for macrolide resistance [17, 18]. The prevalence of macrolide resistance has been shown to decline after stopping azithromycin distributions [19]. Although macrolides are not the most commonly used antibiotic class in settings where trachoma is endemic [20], they are important first-line treatments for many common infections. Early cessation of mass azithromycin distributions in areas that had previously received repeated rounds of treatment may result in reduction of macrolide resistance and would represent an antibiotic-sparing approach to trachoma control.

A current limitation in trachoma surveillance, especially in districts that are nearing control, is indicators for monitoring prevalence and recrudescence. The clinical sign of trachoma TF is easily measured in the field, but it is a lagging indicator of infection and its correlation with infection prevalence wanes in the presence of azithromycin distribution [4, 21, 22]. Measurement of the prevalence of infection requires collection of conjunctival swabs, a cold chain for transport, and laboratory facilities to identify *C. trachomatis* using PCR, which is not feasible for many trachoma programs. Furthermore, measurement of infection provides information on the prevalence of ocular *C. trachomatis* at a single point in time. The antigens Pgp3 and CT694 are *C. trachomatis*-specific and can be more easily measured via collection of dried blood spots and do not require a cold chain, which may facilitate their use in surveillance [9, 23, 24]. Serological measurements of trachoma may provide additional information beyond point prevalence because they allow for estimation of seroconversion rates which can provide insight into transmission. IgG is a durable response, and thus integrates a child’s infection experience over multiple months or years, rather than measuring acute infection as with ocular chlamydia prevalence. We will measure serological outcomes in children, which we expect to provide important information about seroepidemiology in low prevalence settings as well as additional information about transmission of infection in the absence of azithromycin distribution.

Potential limitations of our study design include generalizability and statistical power. This study is being implemented in Maradi, Niger, a region that has received multiple rounds of mass azithromycin distribution. In districts with similar TF prevalence but different trachoma program implementation histories (e.g., fewer or no rounds of mass azithromycin distribution), stopping or not implementing mass azithromycin distribution may have different implications than in areas that have been treated for many years. ARRET is designed to be simple and easily implementable, and implementation of the ARRET study in additional settings is possible and would help allay concerns about generalizability in settings with different trachoma program histories. For statistical power and resource purposes, the unit of randomization in ARRET is the individual community or village, rather than a district or evaluation unit. While this design choice was made to maximize the number of randomization units given fixed resources for trial implementation, trachoma program decisions are made at the district or evaluation unit level. However, inclusion criteria for the current trial are based on district-level TF prevalence rather than community-level, and we anticipate that results of the trial will be generalizable to larger units.

We anticipate that the ARRET trial will provide evidence of whether mass azithromycin distribution can be discontinued in districts with prevalence above the threshold for trachoma control. This evidence will be useful for trachoma control programs as they near the endgame, where more districts are meeting control criteria and focus is shifting to high prevalence areas that have persistent trachoma despite many years of intervention. Early stopping of azithromycin distribution would allow both for resource allocation to the hardest hit areas as well as reduction in antibiotic selection pressure that can lead to increases in antimicrobial resistance.

## Data Availability

No data are presented in the current report. Upon completion of the trial, de-identified data will be made available in aggregate (at the randomization unit level) to interested parties upon reasonable request to the corresponding author of the primary trial manuscript and with applicable institutional review board approval.

## Abbreviations

DSMC: Data and safety monitoring committee
PCR: Polymerase chain reaction
TF: Trachomatous inflammation—follicular
TI: Trachomatous inflammation—intense
WHO: World Health Organization
